# Comparison of multiple imputation and other methods for the analysis of imputed genotypes

**DOI:** 10.1186/s12864-023-09415-0

**Published:** 2023-06-06

**Authors:** Paul L. Auer, Gao Wang, Guangyou Li, Andrew T. DeWan, Suzanne M. Leal

**Affiliations:** 1 Division of Biostatistics, Institute for Health & Equity, and Cancer Center, Medical College of Wisconsin, Milwaukee, WI 53226 USA; 2 Center for Statistical Genetics, Gertrude H. Sergievsky Center, and the Department of Neurology, Columbia University Medical Center, New York, NY USA; 3 Department of Chronic Disease Epidemiology and Center for Perinatal, Pediatric and Environmental Epidemiology, Yale School of Public Health, Yale University, New Haven, CT USA; 4 Taub Institute for Alzheimer’s Disease and the Aging Brain, Columbia University Medical Center, New York, NY USA

**Keywords:** GWAS, Assocation testing, Multiple imputation

## Abstract

**Background:**

Analysis of imputed genotypes is an important and routine component of genome-wide association studies and the increasing size of imputation reference panels has facilitated the ability to impute and test low-frequency variants for associations. In the context of genotype imputation, the true genotype is unknown and genotypes are inferred with uncertainty using statistical models. Here, we present a novel method for integrating imputation uncertainty into statistical association tests using a fully conditional multiple imputation (MI) approach which is implemented using the Substantive Model Compatible Fully Conditional Specification (SMCFCS). We compared the performance of this method to an unconditional MI and two additional approaches that have been shown to demonstrate excellent performance: regression with dosages and a mixture of regression models (MRM).

**Results:**

Our simulations considered a range of allele frequencies and imputation qualities based on data from the UK Biobank. We found that the unconditional MI was computationally costly and overly conservative across a wide range of settings. Analyzing data with Dosage, MRM, or MI SMCFCS resulted in greater power, including for low frequency variants, compared to unconditional MI while effectively controlling type I error rates. MRM andl MI SMCFCS are both more computationally intensive then using Dosage.

**Conclusions:**

The unconditional MI approach for association testing is overly conservative and we do not recommend its use in the context of imputed genotypes. Given its performance, speed, and ease of implementation, we recommend using Dosage for imputed genotypes with MAF $\ge$ 0.001 and Rsq $\ge$ 0.3.

**Supplementary Information:**

The online version contains supplementary material available at 10.1186/s12864-023-09415-0.

## Background

Genotype imputation has transformed the conduct of genome-wide association studies (GWAS). By imputing unobserved genotypes into sample sets that have relatively limited coverage of variants across the genome, contemporary GWAS can now query tens of millions of genetic variants in a single study. There is a mature literature on methodologies for imputation of genotype values [1–3]. To improve imputation quality, recent efforts have focused on increasing the size and diversity of imputation reference panels [4–6] and providing fast, user-friendly, publicly available imputation services [5, 7]. As these resources have expanded and flourished, it is now common to carry out GWAS of low-frequency variation [i.e., genetic variants with minor allele frequencies (MAF) between 0.001 and 0.01] in addition to assaying common variants (MAF $\ge$ 0.01). Although a large number of methods have been developed to impute genotype data [3], the number of statistical methods to analyze associations between phenotypes and imputed genotypes are limited [8–11].

Imputation is stochastic in nature, and therefore imputed genotypes are not perfect proxies for observed values. Standard imputation software outputs posterior probabilities of each possible genotype along with metrics of the confidence with which a genotype has been imputed. Many of the statistical methodologies for analyzing associations in this context have focused on ways to integrate uncertainty in genotype imputations, whether through Bayesian [8] or frequentist [10] frameworks. Others have considered genotype imputation in the context of simultaneous multi-trait modelling by incorporating posterior probabilities as weights in a Generalized Estimating Equation (GEE) framework [12]. Comparisons between methods have shown that for common genetic variants the simple approach of taking the expectation across posterior probabilities (i.e., “Dosage”) provides a fast and powerful solution [10, 11]. However, it remains unclear how well these methods perform for low-frequency variants.

Here, we show that the computationally expensive unconditional implementation of MI (described in [9]) results in overly conservative test statistics for low-frequency variants and variants with poor imputation quality. By recasting genotype imputation as a measurement error problem, we propose a fully conditional MI procedure using the Substantive Model Compatible Fully Conditional Specification (SMCFCS) [13] that leverages both the dosage and the best guess genotype. We show that MI SMCFCS provides proper type I error control and power similar to other well-established frequentist approaches. Using data from the UK Biobank, we explore the performance of different methods [Dosage, MI SMCFCS, mixture of regressions models (MRM), and Unconditional MI] in a regression framework, in terms of type I error control and statistical power, across a range of settings including: binary and quantitative traits; low-frequency and common variation; and imputation quality ranging from high to low. Finally, we demonstrate the performance of these methods on a known locus for circulating triglyceride (TG) levels that contains multiple, independently associated rare-variants of variable imputation quality.

## Results

### Type I error simulation results

**Fig. 1:**
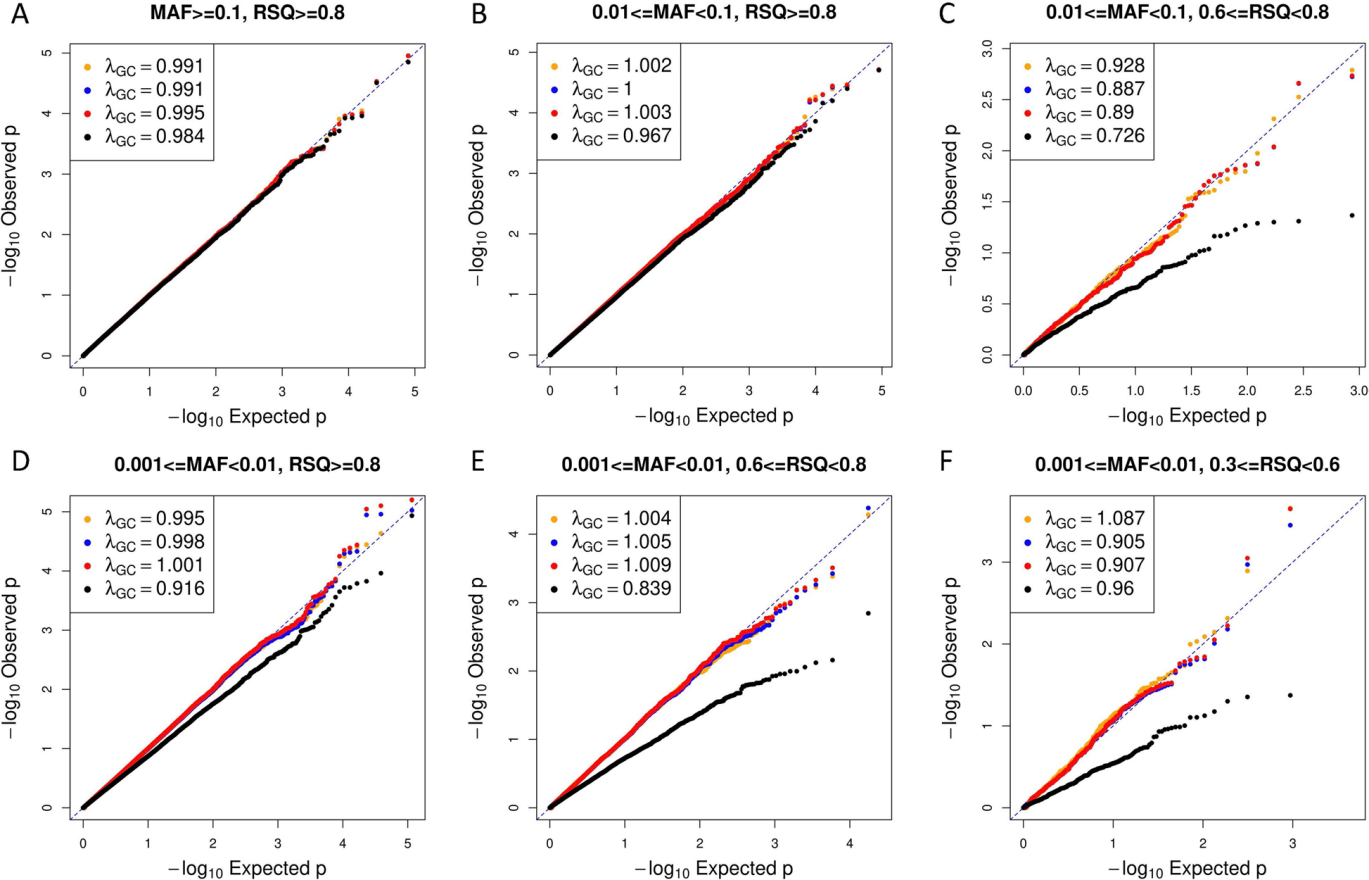
Quantile–Quantile plots of p-values obtained for a binary phenotype with *n* = 50,000 samples and an underlying disease prevalence of 0.5. P-values for Unconditional MI are shown in black, Dosage in blue, MI SMCFCS in orange, and MRM in red. A variety of variant frequencies and Rsq scores were evaluated. Genomic control lambdas for each test are included in the plot

We first assessed type I error control through large-scale simulations (see Methods for details). We compared the type I error control for association testing with Dosage, MI SMCFCS, MRM, and Unconditional MI (see Methods). We simulated phenotypes that were uncorrelated to the imputed genotype values (data generated under the null). Under all scenarios for binary phenotypes, type I error was effectively controlled for all methods (Fig. 1).

For variants with MAF $\ge$ 0.01, all methods displayed well-calibrated p-values for variants with good imputation quality (Rsq $\ge$ 0.8, Fig. 1A, B). For variants with 0.01 $\le$ MAF < 0.1 and 0.6 $\le$ Rsq < 0.8, Unconditional MI was overly conservative [Fig. 1C, genomic control (GC) lambda = 0.726] while Dosage, MI SMCFCS, and MRM showed well-calibrated *p*-values (Fig. 1C). For low-frequency variants, (i.e., those with 0.001 $\le$ MAF < 0.01) Unconditional MI was overly conservative across the range of imputation qualities (Fig. 1D-F) whereas the quantile–quantile plots for Dosage, MI SMCFCS, and MRM were all well-behaved. We observed the same pattern of results for binary traits with *n* = 20,000 (Figure S1), i.e., Dosage, MI SMCFCS, and MRM displayed well-calibrated p-values under all settings but Unconditional MI displayed overly conservative results when Rsq < 0.8 and when MAF < 0.01. For quantitative traits, the same exact pattern held as for binary traits for *n* = 20,000 (Figure S2) and *n* = 50,000 (Figure S3).

### Statistical Power simulation results

To compare the statistical power of Dosage, MI SMCFCS, MRM, and Unconditional MI approaches, we ran simulations for both binary and quantitative phenotypes using a range of different effect sizes, and assuming that the directly genotyped values (as ascertained via WES) represented the true genotypes (see Methods). Table 1 shows the power of all four methods with ~ 10,000 cases and ~ 40,000 controls.

**Table 1:** Simulated power with a binary trait and *n* = 50,000 observations with an underlying disease prevalence of 0.2

**Odds ratios**	**Method**	**MAF **≥ **0.1** **Rsq** ≥ **0.8**	**0.01** ≥ **MAF < 0.1** **Rsq** ≥ **0.8**	**0.01** ≤ **MAF < 0.1** **0.6** ≤ **Rsq < 0.8**	**0.001** ≤ **MAF < 0.01** **0.3** ≤ **Rsq < 0.6**	**0.001** ≤ **MAF < 0.01** **0.6** ≤ **Rsq < 0.8**	**0.001** ≤ **MAF < 0.01** **Rsq** ≥ **0.8**
1–1.2	MI SMCFCS	0.42	0.05	0	0	0	0
	Dosage	0.42	0.05	0	0	0	0
	MRM	0.43	0.05	0	0	0	0
	U-MI^a^	0.42	0.04	0	0	0	0
1.2–1.4	MI SMCFCS	1	0.6	0.2	0	0	0
	Dosage	1	0.6	0.2	0	0	0
	MRM	1	0.61	0.23	0	0	0
	U-MI^a^	1	0.57	0.07	0	0	0
1.4–1.6	MI SMCFCS	1	0.94	0.68	0.01	0.02	0.09
	Dosage	1	0.94	0.66	0.01	0.02	0.08
	MRM	1	0.95	0.71	0.02	0.03	0.10
	U-MI^a^	1	0.91	0.44	0	0	0.05
1.6–1.8	MI SMCFCS	1	1	0.98	0.03	0.14	0.32
	Dosage	1	1	0.98	0.04	0.14	0.31
	MRM	1	1	0.98	0.06	0.16	0.34
	U-MI^a^	1	0.99	0.80	0	0.03	0.22
1.8–2.0	MI SMCFCS	1	1	1	0.13	0.27	0.53
	Dosage	1	1	1	0.18	0.26	0.51
	MRM	1	1	1	0.19	0.29	0.54
	U-MI^a^	1	1	0.92	0.03	0.10	0.42
2.0 – 3.0	MI SMCFCS	1	1	1	0.63	0.81	0.91
	Dosage	1	1	1	0.66	0.80	0.90
	MRM	1	1	1	0.66	0.80	0.91
	U-MI^a^	1	1	1	0.29	0.61	0.85

^a^U-MI = Unconditional MI

All four methods had very similar power when Rsq $\ge$ 0.8 and MAF $\ge$ 0.1. As MAF decreased to 0.01 $\le$ MAF < 0.1 while retaining Rsq $\ge$ 0.8, Unconditional MI started to show slightly decreased power compared to Dosage, MI SMCFCS, and MRM. This drop-off in power for Unconditional MI became more apparent with 0.001 $\le$ MAF < 0.01 and Rsq $\ge$ 0.8. With 0.01 $\le$ MAF < 0.1 and 0.6 $\le$ Rsq < 0.8, the power of Unconditional MI was often at least 10% lower than the power of Dosage, MI SMCFCS, and MRM. With 0.001 $\le$ MAF < 0.01 and 0.3 $\le$ Rsq < 0.8, the power of Unconditional MI was often < 50% than that of Dosage, MI SMCFCS, and MRM. Under all of these settings, Dosage, MI SMCFCS, and MRM displayed remarkably similar power. Generally, the only context in which Unconditional MI was similarly powered to the other methods was when Rsq $\ge$ 0.8 and MAF $\ge$ 0.1. Under all other settings, Unconditional MI was under-powered compared to the other three methods. As we changed the disease prevalence to 0.1 (~ 5,000 cases and ~ 45,000 controls) (Table S1), we observed the same pattern of results. With Unconditional MI becoming increasingly under-powered as MAF and Rsq decreased compared to the other methods which had similiar power. The same was true with ~ 15,000 cases and ~ 35,000 controls (Table S2) were analyzed. For quantitative traits with *n* = 20,000 samples (Table S3), we observed that Unconditional MI was under-powered as MAF decreased from 0.1 and Rsq decreased from 0.8, with the drop-off in power similar to that observed for binary traits. This pattern did not change when we increased the sample size to *n* = 50,000 samples for quantitative traits (Table S4). Consistent with our intuition, all methods lost power as MAF and Rsq decreased under all simulation settings for both binary and quantitative traits.

### Comparison of compute times

In addition to comparing the type I error and power of these methods, we also analyzed the computational burden of each method. All methods were implemented in R version 4.1.1 on a virtual machine with 16vCPU, 96 Gb of memory and an Intel Xeon Gold 6240R processor. We considered a single SNP with MAF = 0.1 and a range of sample sizes and effect sizes in our analyses of both quantitative and binary traits. Not suprisingly, Dosage was by far the fastest method (Tables S5 and S6); the Unconditional MI was at least an order of magnitude slower than Dosage, even with a relatively small number of imputation repetitions (M = 5). Both MRM and SMCFCS were orders of magnitude slower than both Dosage and Unconditional MI. The compuational cost of MRM was borne by the optimization of the likelihood function and the SMCFCS procedure runs a full Markov Chain Monte Carlo procedure for each imputation repetition. Our analysis suggests that of these four methods, perhaps only Dosage results in a reasonable computational burden when analyzing millions of variants. Though any method that analyzes each SNP separately can be made to run quickly given access to cloud computing and the embarrassingly parallel nature of the task, but potentially at great monetary expense.

### Data analysis

To compare methods using a real-data example of true positive single nucleotide variant (SNV)-trait associations with rare and poorly imputed variants, we considered three variants (rs76353203, rs138326449, rs140621530) in the *APOC3* gene that are known to be associated with circulating TG levels [14, 15]. We conducted association tests with Dosage, MI SMCFCS, MRM, and Unconditional MI, between rs76353203, rs138326449, and rs140621530 and TG levels using data from the UK Biobank (see Methods). The association results are shown in Table 2. One marker with moderate imputation quality (rs76353203, Rsq = 0.602, MAF = 1.38 × 10^–4^) showed the biggest discrepancy in results with MRM (*p* = 2.2 × 10^–6^), MI SMCFCS (*p* = 1.25 × 10^–6^), and Dosage (*p* = 1.9 × 10^–7^) providing larger signals (i.e., smaller p-values) compared to Unconditional MI (*p* = 3.2 × 10^–3^). Even for a rare variant with higher imputation quality (rs138326449, Rsq = 0.866, MAF = 1.50 × 10^–3^), again Unconditional MI displayed the weakest signal [*p* = 1.21 × 10^–31^ (Unconditional MI), *p* = 7.17 × 10^–47^ (MI SMCFCS), *p* = 4.22 × 10^–47^ (MRM), and *p* = 1.62 × 10^–47^ (Dosage)].

**Table 2:** Association results from the MI SMCFCS, Unconditional MI, Dosage, and MRM for the three imputed TG-associated *APOC3* SNVs

Variant	MAF	Rsq	MI SMCFCS	Dosage	MRM	Unconditional MI
rs76353203 (p.Arg19*) R19X	1.38 × 10^–4^	0.602	1.25 × 10^–6^	1.9 × 10^–7^	2.2 × 10^–6^	3.2 × 10^–3^
rs138326449 (c.55 + 1G > A) IVS2 + 1G > A	1.50 × 10^–3^	0.866	7.17 × 10^–47^	1.62 × 10^–47^	4.22 × 10^–47^	1.21 × 10^–31^
rs140621530 (c179 + 1G > T) IVS3 + 1G > T	2.88 × 10^–5^	0.392	0.619	0.223	0.861	0.859

## Discussion

In this study, we compared the performance of Unconditional MI to three different methods, including a new conditional MI, i.e., MI SMCFCS, for conducting association testing with imputed genotype data. The Unconditional MI method as described in Palmer and Pe’er 2016 [9] performed well for common, well-imputed variants. But we observed a noticeable drop-off in performance (i.e., overly conservative *p*-values) as imputation quality decreased. For the first time, we present a different approach to multiple imputation in the context of genetic association studies with imputed genotypes. By conditioning on the outcome variable, we implemented the SMCFCS approach of Grey [16] and Keogh and White [17] to perform MI. In so doing, we show that this enhanced multiple imputation strategy outperforms the Unconditional MI approach from Palmer and Pe’er 2016 with results similar to using Dosage or MRM. The results from our simulations did not substantively change when we increased the number of rounds of multiple imputation from M = 5 to M = 20 and M = 50 (data not shown), suggesting that the conservative results we observed were attributable to the method itself rather than the details of our implementation.

Our conclusions add to the results from Palmer and Pe’er 2016 regarding the use of Unconditional MI in imputation-based GWAS. Palmer and Pe’er 2016 focused on common variants (MAF > 0.05) with high confidence imputation scores and compared the relative ranking of SNVs under both null and alternative hypotheses. Under these conditions, they found that Unconditional MI properly ranks variants more successfully than other methods. But their study did not perform standard type I error or power simulations as was done here. Our simulations clearly demonstrate the overly conservative performance of Unconditional MI under most settings, especially for low-frequency variants (MAF $\le$ 0.01) with poor imputation quality (Rsq $<$ 0.8). When analyzing true positive associations with less frequent and poor imputation quality variants in the *APOC3* gene, we also show that Unconditional MI provides the weakest association signal compared to Dosage, MI SMCFCS, and MRM.

Perhaps unsurprisingly, Rubin 1996 [18] foresaw the performance of the Palmer and Pe’er 2016 implementation of Unconditional MI in this context. Generally, when an outcome variable Y is left out of the imputation scheme for an independent variable G, the imputation is considered “improper” and “generally leads to biased estimation and invalid inference. For example, if Y is correlated to G but not used to multiply-impute G, then the multiply-imputed data set will yield estimates of the YG correlation biased towards zero.” This is in fact, precisely what we observed with the overly conservative performance of the Unconditional MI. By using the outcome variable to perform a “proper” fully conditional imputation of G (in the SMCFCS framework), we have overcome this issue and provided a valid and rigorous multiple imputation method that performs well compared to other more well-established approaches (i.e., Dosage and MRM).

Our study was limited to comparisons of four methods (Unconditional MI, Dosage, MRM, MI SMCFCS) as we did not include the methods implemented in SNPTEST (i.e., the Score Test, the EM-algorithm, or Bayesian modeling). However, the focus of our work was to evaluate the performance of multiple imputation methods and for this purpose, we believe that Dosage and MRM were sufficient for our comparisons. As in Zheng et al. [10], we found that using Dosage was an efficient and powerful approach under most settings. Our implementation of the MRM focused on the 1 degree of freedom test with an additive genetic model. In this setting, we found that MRM and Dosage performed remarkably similarly and in contrast to Zheng et al. [10], we did not explore the performance of these methods in the context of small sample sizes (*n* = 50) with large effects. Our implementation of MRM and MI SMCFCS was also very computationally expensive, without any gain in performance under realistic simulation settings. Overall, we observed that for variants with MAF $\ge$ 0.001 and reasonable imputation quality (Rsq $\ge$ 0.3), using Dosage provides a fast, robust, and powerful approach. Our type I error simulations support this recommendation, as using Dosage effectively controlled the rate of false positives. However, we do note that for rare variants (e.g. MAF < 0.001) or variants with low imputation quality (Rsq < 0.3), power will likely be low without extremely large sample sizes and we did not investigate this class of variants here. For very rare variants (MAF < 0.001) and/or variants of poor imputation quality (Rsq < 0.3), more research is needed to assess the performance of different methods for handling imputation uncertainty in association testing. As imputation reference panels continue to expand [6], very rare variants may become imputable with reasonable confidence. Future research is needed to integrate uncertainty (beyond using Dosage) into aggregate rare variant association tests (e.g., CMC [19] or SKAT [20]).

## Conclusions

We compared the performance of four different methods for incoporating imputation uncertainty into statistical tests of association: Dosage, MRM, unconditional MI, and conditional MI. The Dosage, MRM, and unconditional MI approaches all performed similarly across a range of MAFs and imputation qualities and in a real data analysis. However, we found that the unconditional MI approach was overly conservative for variants with low imputation quality (Rsq < 0.8) or low frequency (MAF < 0.01) and we do not recommend its use in association testing of imputed genotypes.

## Methods

### Dosage, MI, and MRM

We evaluated four different approaches to analyzing imputed genotypes for associations with both binary and quantitative traits; namely Dosage, MI SMCFCS, MRM, and Unconditional MI. Briefly, we let ${y}_{i}$ denote the phenotypic value for the ${i}^{th}$ individual and ${G}_{ij}$ represent the true genotype for the ${i}^{th}$ individual at the ${j}^{th}$ genetic marker. In an imputation-based association study, ${G}_{ij}$ is not directly observed but instead the posterior probabilities of the three genotypes are output from standard imputation software. We denote the following genotype probabilities for the ${i}^{th}$ individual at the ${j}^{th}$ marker: reference allele homozygote ${p}_{0ij}$ heterozyogote ${p}_{1ij}$, and alternative allele homozygote ${p}_{2ij}$, respectively.

To model the association with Dosage, we take the expectation across the posterior probabilities where the dosage for each ${i}^{th}$ individual at the ${j}^{th}$ marker is: ${D}_{ij}={p}_{1ij}+2{p}_{2ij}$. For quantitative traits we apply a linear regression model: ${y}_{i}={\beta }_{0}+{\beta }_{1}{D}_{ij}+{\epsilon }_{i}$, where ${\epsilon }_{i}\sim N\left(0,{\sigma }^{2}\right)$, and for binary traits a logistic regression model with $log\frac{{\pi }_{i}}{(1-{\pi }_{i})}={\beta }_{0}+{\beta }_{1}{D}_{ij}$, where ${\pi }_{i}=P({y}_{i}=1)$. The Dosage model incorporates uncertainty into the association test because it differentiates genotypes that were imputed with high confidence from those that were imputed with low confidence.

To model the association using Unconditional MI, we use a random number generator to impute genotypes based on their posterior probabilities. Let ${\widetilde{G}}_{ij}$ denote the corresponding imputed genotype value where $P\left({\widetilde{G}}_{ij}=0\right)= {p}_{0ij}, P\left({\widetilde{G}}_{ij}=1\right)= {p}_{1ij}, \mathrm{and} P\left({\widetilde{G}}_{ij}=2\right)= {p}_{2ij} .$ We then run either a linear model ${y}_{i}={\beta }_{0}+{\beta }_{1}{\widetilde{G}}_{ij}+{\epsilon }_{i}$ or a logistic regression model$log\frac{{\pi }_{i}}{(1-{\pi }_{i})}={\beta }_{0}+{\beta }_{1}{\widetilde{G}}_{ij}$. This procedure is repeated M times (M = 5 in our simulations) to produce M estimates of ${\widehat{\beta }}_{1}$ and their associated standard errors${SE(\widehat{\beta }}_{1})$. The estimates and standard errors are then combined using Rubin’s rules [9, 21] to produce a p-value that assesses the genetic association. The Unconditional MI approach incorporates uncertainty by sampling over possible instances of the genotype. The main distinction between Unconditional MI and Dosage is that the standard erros in the Unconditional MI approach directly accounts for uncertainty, whereas the standard errors in the Dosage model assume that the dosages are known without error.

Unconditional MI does not condition on any other covariates or outcome variables. In order to improve on this unconditional imputation, we reframe the analysis of imputed genotypes as a measurement error problem and can therefore use the value of the observed outcomes to provide more accurate imputations. In this framework, we are interested in making inferences about the assocation between *G* and *Y*, but we do not observe G directly. Instead, we observe a version of *G* subject to error, denoted by our dosage variable *D*. In the following, we assume classical measurement error, i.e., ${D}_{ij}={G}_{ij}+{\eta }_{ij}$, where the error terms ${\eta }_{ij}$ have mean zero and constant variance and are uncorrelated with *Y* and *G*. In this setting, we can consider a conditional imputation of *G* by the following typical imputation model: ${G}_{ij}={\gamma }_{0}+{\gamma }_{1}{D}_{ij}+{\gamma }_{2}{Y}_{i}+{e}_{ij}$. As in Keogh and White [17], the M^th^ imputed value for ${G}_{ij}$ is taken from a distribution *f*, with mean =${E(G}_{ij} \left| {D}_{ij}, {Y}_{i}\right)$ and variance = ${Var(G}_{ij} \left| {D}_{ij}, {Y}_{i}\right)$. So obtaining imputed values amounts to estimating *f* using only observed data, as outlined in Keogh and Bartlett 2019 [22] and developed in Gray 2018 [16]. Briefly, *f* is defined as a posterior distribution given a likelihood and a prior distribution. Model parameters ${\theta }^{*}$ are drawn from their approximate posterior distribution and then imputed values ${G}^{C}$ are drawn from $f(G|{\theta }^{*},Y,D)$. A rejection rule is used to determine whether ${G}^{C}$ is accepted as a value from $f(G|\theta ,Y,D)$. These steps are repeated for every individual. Finally, the algorithm is repeated iteratively until the imputed G values converge to a stationary distribution. The last cycle of the imputed values are used as the final imputed values for G. This SMCFCS model is implemented in the SMCFCS R-package (https://github.com/jwb133/smcfcs).

In order to estimate *f* as in the above, we must assume that there are two noisy measurements for *G*. Here, we use the genotype dosage *D* as well as the best-guess genotypes based on the imputation posterior probabilities, denoted W. Our rationale for using the best-guess genotype as our second noisy measurement was twofold: (i) it is a convenient measurement of *G* that is readily available from genotype imputation software; and (ii) when ${D}_{ij}$ and ${W}_{ij}$ are very close to each other, then we conclude that the imputation was performed with high confidence (i.e., one of ${p}_{0ij}$, ${p}_{1ij}$, or ${p}_{2ij}$ is close to 1), the variance of *f* would be relatively small, and the multiple imputations of ${G}_{ij}$ would have relatively low variance, whereas when ${D}_{ij}$ and ${W}_{ij}$ are dissimilar, then we conclude that the imputation was not performed with high confidence (i.e., none of ${p}_{0ij}$, ${p}_{1ij}$, or ${p}_{2ij}$ are close to 1), the variance of *f* would be relatively large, and the multiple imputations of ${G}_{ij}$ would have relatively high variance. Though *D* and *W* should ideally be independent (in our case they are clearly dependent), the SMCFCS procedure is apparently robust in this context as it provides reasonable results (see Results). Importantly, the SMCFCS methodology allows for non-linear relationships between *G* and *Y*, for instance as modeled in a logistic regression or a Cox regression.

To model the association with MRM, we follow the approach detailed in Zheng et al. [10] for quantitative traits. The MRM model directly incorporates imputation uncertainty by including the imputation posterior probabilities into the likelihood function, rather than taking the expectation (Dosage) or sampling over possible values of the genotypes (Unconditional MI and SMCFCS). Specifically, we test for association via a likelihood ratio test, where the log-likelihood function is: $ll\left({\beta }_{0},{\beta }_{1}\right)= \sum_{i=1}^{n}\mathrm{log}(f\left({y}_{i}\right))$, and $f\left({y}_{i}\right)= \frac{1}{\sqrt{2\pi {\sigma }^{2}}}({p}_{0ij}{e}^{\frac{{-\left({y}_{i}-{\beta }_{0}\right)}^{2}}{2{\sigma }^{2}}}+ {p}_{1ij}{e}^{\frac{{-\left({y}_{i}-{\beta }_{0}-{\beta }_{1}\right)}^{2}}{2{\sigma }^{2}}}+{p}_{2ij}{e}^{\frac{{-\left({y}_{i}-{\beta }_{0}-2{\beta }_{1}\right)}^{2}}{2{\sigma }^{2}}}$).

To model the association with MRM and a binary trait we replace the log-likelihood function with: $ll\left({\beta }_{0},{\beta }_{1}\right)= \sum_{i=1}^{n}\mathrm{log}(f\left({y}_{i}\right))$, and $f\left({y}_{i}\right)= {p}_{0ij}{\pi }_{i0}^{{y}_{i}}{(1-{\pi }_{i0})}^{1-{y}_{i}} + { p}_{1ij}{\pi }_{i1}^{{y}_{i}}{(1-{\pi }_{i1})}^{1-{y}_{i}} + {p}_{2ij}{\pi }_{i2}^{{y}_{i}}{(1-{\pi }_{i2})}^{1-{y}_{i}}$, where${\pi }_{i0}=\frac{1}{1+{e}^{(-{\beta }_{0})}}$, ${\pi }_{i1}=\frac{1}{1+{e}^{(-{\beta }_{0}-{\beta }_{1})}}$ , and ${\pi }_{i2}=\frac{1}{1+{e}^{(-{\beta }_{0}-2{\beta }_{1})}}$.

As in Zheng et al. [10] we implemented these approaches in the R statistical computing environment. For the MRM, we maximized the log-likelihoods via a modified Broyden–Fletcher–Goldfarb–Shanno (BFGS) quasi-Newton method implemented in the optim() function [23] in R.

### UK biobank data

To evaluate the performance of Dosage, MI SMCFCS, MRM, and Unconditional MI we based our simulations and real data analyses on genotypes and phenotypes from the UK Biobank study [24]. The UK Biobank recruited 502,639 participants (aged 37–73 years) from 22 assessment centers across the UK between 2007 and 2010. All participants gave written informed consent before enrollment in the study, which was conducted in accordance with the principles of the Declaration of Helsinki. As described in Bycroft et al. [25], participants were genotypes on one of two very similar (95% of the marker content is the same) genotyping arrays: the UK BiLEVE Axiom Array by (807,411 markers) or the Applied Biosystems UK Biobank Axiom Array (825,927 markers). The resulting genotypes underwent stringent sample-level and marker-level quality control filters. Haplotypes were then estimated via SHAPEIT3 [26] and samples were imputed to both the Haplotype Reference Consortium [5] reference panel as well as a combined UK10K 1000 Genomes reference panel using IMPUTE4 [2]. A total of 93 million autosomal markers were imputed in 487,442 individuals. In addition to the imputed genetic data, the UK Biobank has also generated whole-exome sequencing (WES) data on ~ 450,000 participants. Over 10 million variants were observed in the WES target region, with the vast majority of variants having MAF < 1% [27]. Non-fasting venous blood sampling was also conducted, and biochemistry measures were performed at a dedicated central laboratory between 2014 and 2017, that included TG levels [28]. For the simulations, to estimate type I error and power, we used 50,000 unrelated participants from the UK Biobank who were designated as having white British ancestry in Bycroft et al. [25].

### Type I error simulation methods

To evaluate type I error control and power, we treated the WES-based genotypes as the “truth” for simulating phenotypes and analyzed the data using the imputed genetic data. So that our results did not reflect any systematic, UK Biobank specific inaccuracies in imputation, we compared the true Rsq (i.e., the squared correlation between the WES-based genotype and the imputation based dosage) with the imputation Rsq (i.e., the estimated Rsq calculated only from dosages). We calculated the percent difference between the imputation Rsq and the true Rsq and only considered variants in our simulations for which the percent difference was less than 20%. This resulted in a total of 181,292 exonic variant from chromosomes 1–22 with MAF $\ge$ 0.001 and imputation Rsq $\ge$ 0.3 (Table 3).

**Table 3:** The numbers of markers analyzed in the type I error and power simulations across MAF and imputation Rsq bins

Imputation Rsq	MAF	Number of variants
[0.8, 1]	[0.1, 0.5]	43,837
[0.8, 1]	[0.01, 0.1)	58,157
[0.8, 1]	[0.001, 0.01)	68,041
[0.6, 0.8)	[0.01, 0.1)	463
[0.6, 0.8)	[0.001, 0.01)	10,247
[0.3, 0.6)	[0.001, 0.01)	547

To simulate quantitative traits unrelated to these genetic variants, we drew phenotype values from a Normal ($\mu =0,{\sigma }^{2}=1$) distribution for each marker. To simulate null binary traits, we drew phenotype values from a Bernoulli (${\pi }_{i}=0.5$) distribution for each marker (i.e., the equivalent of a prevalence of 0.5). Samples were generated with 20,000 and 50,000 “individuals” for both quantitative and binary traits. Associations were tested using Unconditional MI (with M = 5 rounds of imputation), MI SMCFCS (M = 5), Dosage, and MRM, as described above. To evaluate type I error rates, we created quantile–quantile plots using the *p*-values of the variants in each MAF and Rsq bin from Table 1.

### Statistical power simulation methods

Quantitative traits for the $${i}^{th}$$ individual at the $${j}^{th}$$ genetic marker were simulated from the WES-based genotype ($${G}_{ij})$$ value as $${Y}_{ij}={\beta }_{1}{G}_{ij}+{\epsilon }_{i}$$, where $${\epsilon }_{i}\sim N(0,{\sigma }^{2}=1)$$ and $${\beta }_{1}$$(i.e., the effect size in trait standard deviations) values were randomly drawn from a |$$N(0,{\sigma }^{2}=1)$$| distribution. In this way, each genetic variant was assigned its own unique positively valued effect size. Quantitative traits were simulated for n = 20,000 and n = 50,000 samples from the resulting Normal distributions. Binary traits for the $${i}^{th}$$ individual at the $${j}^{th}$$ genetic marker were simulated from the WES-based genotype ($${G}_{ij})$$ value as well, assuming an underlying disease prevalence of $$\tau$$ = 0.1, 0.2, or 0.3. The probability of disease for the $${i}^{th}$$ individual at the $${j}^{th}$$ genetic marker was modeled as:$${\pi }_{ij}=\frac{{e}^{({\beta }_{0}+{\beta }_{1}{G}_{ij})}}{1+{e}^{({\beta }_{0}+{\beta }_{1}{G}_{ij})}}$$, where$${\beta }_{0}=log\frac{\tau }{1-\tau }$$, and $${\beta }_{1}$$ values were randomly drawn from a |$$N(0,{\sigma }^{2}=1)$$| distribution as well. Similar to the quantitative trait simulations, each genetic variant was assigned its own unique positively valued effect size. To generate binary phenotypes, we drew *n* = 50,000 Bernoulli($${\pi }_{ij})$$ trials. With an underlying disease prevalence of 0.1, 0.2, and 0.3, we simulated three different data sets with *n* = 50,000 resulting in ~ 5,000 cases and ~ 45,000 controls, ~ 10,000 cases and ~ 40,000 controls, and ~ 15,000 cases and ~ 35,000 controls, respectively. Power was evaluated at the genome-wide significance level (α = 5.0 × 10^–8^), i.e., within each MAF, Rsq, and effect size bin, we calculated power as the average number of times that *p*-value < 5 × 10^–8^.

### Analysis of variants in *APOC3* with TG levels

We considered 56,073 unrelated samples of white European ancestry from the UK Biobank with measured TG levels and genetic data. As in Auer et al. [14], we regressed log (TG) levels against age, sex, and the first two genetically derived principal components. The residuals from this model were then tested for association using Dosage, MI SMCFCS, MRM, and Unconditionial MI with the three variants [rs76353203 (p.Arg19*) R19X; rs138326449 (c.55 + 1G > A) IVS2 + 1G > A; and rs140621530 (c179 + 1G > T) IVS3 + 1G > T] in *APOC3*.

## Supplementary Information

**Additional file 1.** 

## Data Availability

Access to the UKBiobank data are granted via the UKBiobank registration: (https://www.ukbiobank.ac.uk/enable-your-research/register).

## Abbreviations

MI: Multiple imputation
MRM: Mixture of regression models
GWAS: Genome-wide association studies
MAF: Minor allele frequency
SMCFCS: Substantive Model Compatible Fully Conditional Specification
TG: Triglyceride
WES: Whole-exome sequencing
