# Supplementary material for: Comparison of multiple imputation and other methods for the analysis of imputed genotypes
Source: BMC Genomics. 2023 Jun 6;24:303. doi: 10.1186/s12864-023-09415-0 (PMC10242917; doi:10.1186/s12864-023-09415-0)
Supplement: Supplementary file 1 — Additional file 1. [file 12864_2023_9415_MOESM1_ESM.docx]

**Supplementary Figures**


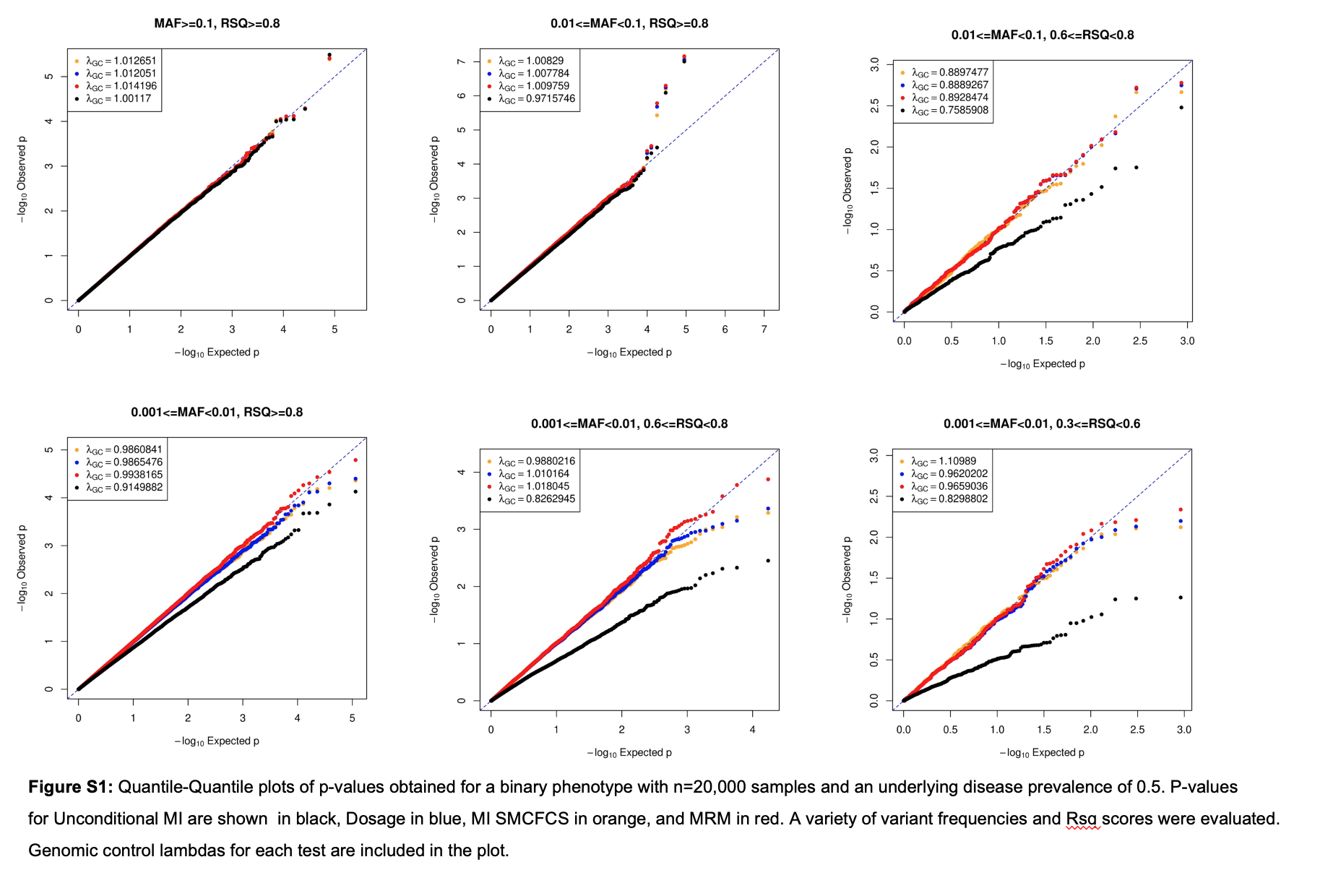


**
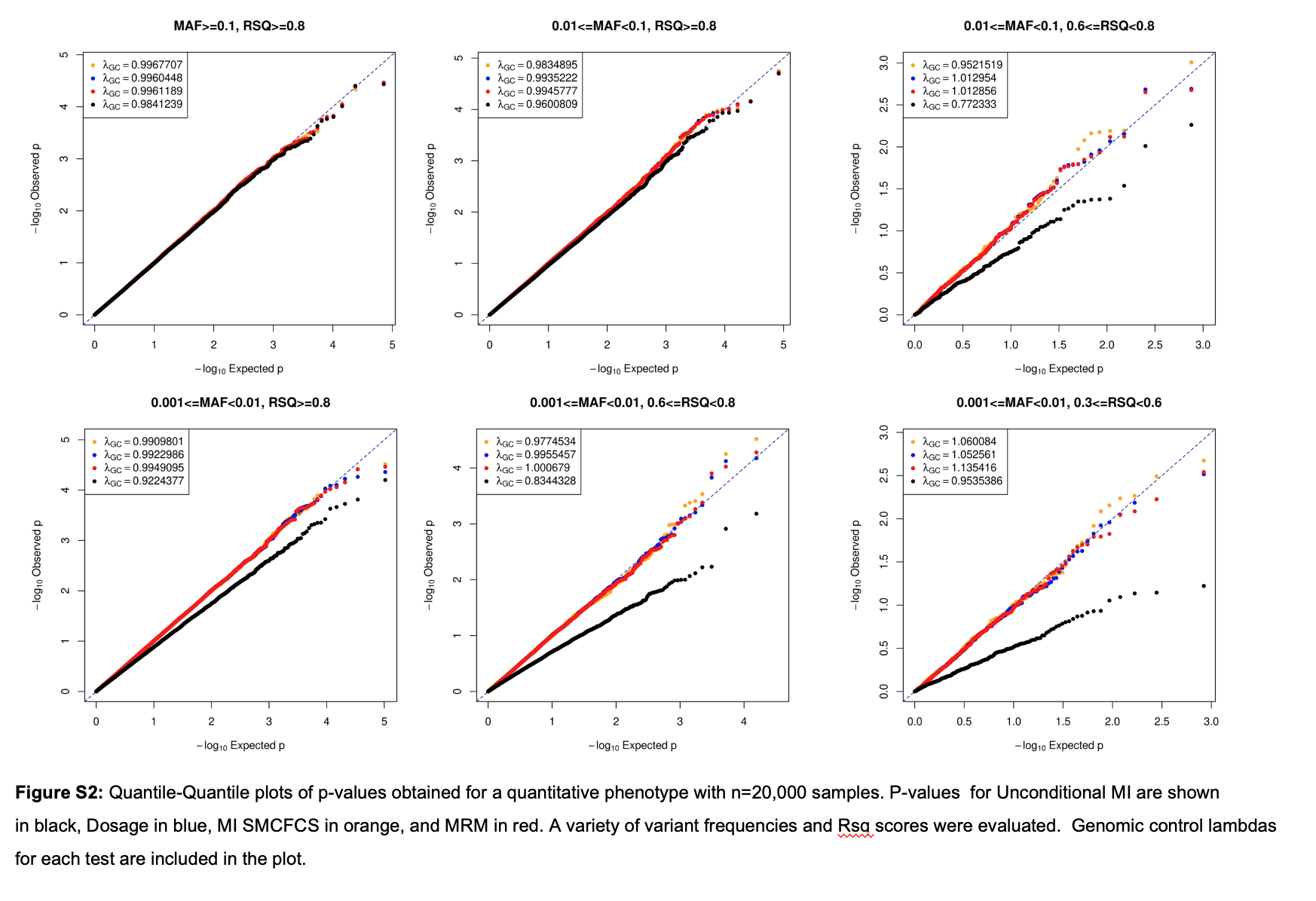
**

**
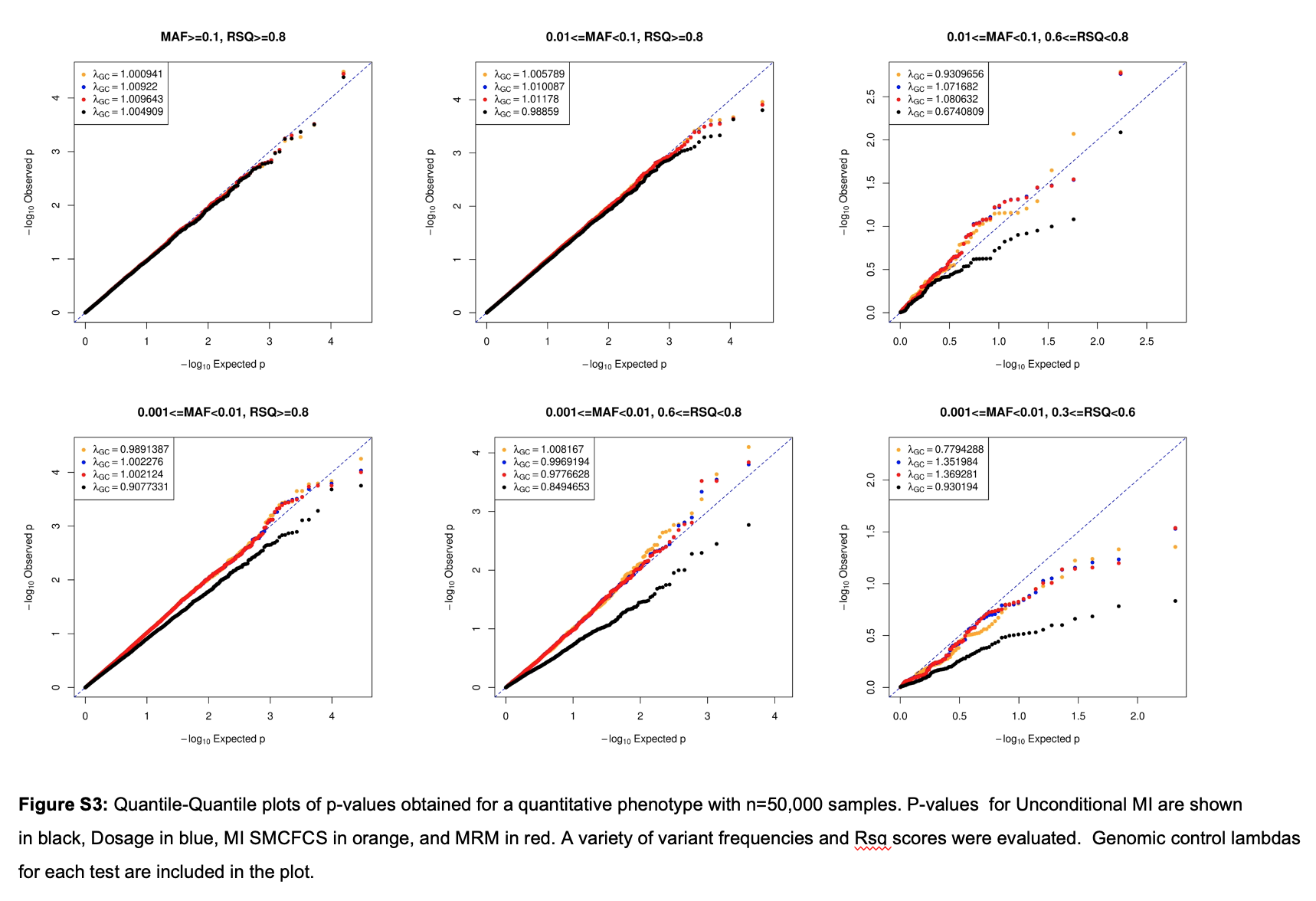
**

**Supplementary Tables**

**Table S1**: Simulated power with a binary trait and n=50,000 observations with an underlying disease prevalence of 0.1.

| **Odds ratios** | **Method** | **MAF**$\boldsymbol{\geq}$**0.1**  **Rsq**$\boldsymbol{\geq}$**0.8** | **0.01**$\boldsymbol{\leq}$**MAF<0.1**  **Rsq**$\boldsymbol{\geq}$**0.8** | **0.01**$\boldsymbol{\leq}$**MAF<0.1**  **0.6**$\boldsymbol{\leq}$**Rsq<0.8** | **0.001**$\boldsymbol{\leq}$**MAF<0.01**  **0.3**$\boldsymbol{\leq}$**Rsq<0.6** | **0.001**$\boldsymbol{\leq}$**MAF<0.01**  **0.6**$\boldsymbol{\leq}$**Rsq<0.8** | **0.001**$\boldsymbol{\leq}$**MAF<0.01**  **Rsq**$\boldsymbol{\geq}$**0.8** |
| --- | --- | --- | --- | --- | --- | --- | --- |
| 1-1.2 | MI SMCFCS  Dosage  MRM  U-MI* | 0.26  0.26  0.27  0.25 | 0.01  0.01  0.01  0.01 | 0  0  0  0 | 0  0  0  0 | 0  0  0  0 | 0  0  0  0 |
| 1.2-1.4 | MI SMCFCS  Dosage  MRM  U-MI* | 0.99  0.99  0.99  0.99 | 0.36  0.36  0.39  0.34 | 0.15  0.15  0.15  0.05 | 0  0  0  0 | 0  0  0  0 | 0  0  0  0 |
| 1.4-1.6 | MI SMCFCS  Dosage  MRM  U-MI* | 1  1  1  1 | 0.79  0.79  0.82  0.75 | 0.43  0.43  0.45  0.16 | 0  0  0  0 | 0  0  0.01  0 | 0.01  0.01  0.02  0.01 |
| 1.6-1.8 | MI SMCFCS  Dosage  MRM  U-MI* | 1  1  1  1 | 0.97  0.97  0.98  0.94 | 0.77  0.78  0.81  0.49 | 0.01  0.01  0.02  0 | 0.04  0.04  0.05  0.01 | 0.12  0.11  0.16  0.07 |
| 1.8-2.0 | MI SMCFCS  Dosage  MRM  U-MI* | 1  1  1  1 | 1  1  1  0.99 | 0.98  0.97  0.98  0.8 | 0.04  0.04  0.05  0 | 0.14  0.13  0.17  0.03 | 0.32  0.3  0.37  0.22 |
| 2.0 – 3.0 | MI SMCFCS  Dosage  MRM  U-MI* | 1  1  1  1 | 1  1  1  1 | 1  1  1  0.98 | 0.54  0.53  0.57  0.22 | 0.73  0.71  0.75  0.52 | 0.85  0.83  0.86  0.77 |
| *U-MI = Unconditional MI | | | | | | | |

**Table S2**: Simulated power with a binary trait and n=50,000 observations with an underlying disease prevalence of 0.3.

| **Odds ratios** | **Method** | **MAF**$\boldsymbol{\geq}$**0.1**  **Rsq**$\boldsymbol{\geq}$**0.8** | **0.01**$\boldsymbol{\leq}$**MAF<0.1**  **Rsq**$\boldsymbol{\geq}$**0.8** | **0.01**$\boldsymbol{\leq}$**MAF<0.1**  **0.6**$\boldsymbol{\leq}$**Rsq<0.8** | **0.001**$\boldsymbol{\leq}$**MAF<0.01**  **0.3**$\boldsymbol{\leq}$**Rsq<0.6** | **0.001**$\boldsymbol{\leq}$**MAF<0.01**  **0.6**$\boldsymbol{\leq}$**Rsq<0.8** | **0.001**$\boldsymbol{\leq}$**MAF<0.01**  **Rsq**$\boldsymbol{\geq}$**0.8** |
| --- | --- | --- | --- | --- | --- | --- | --- |
| 1-1.2 | MI SMCFCS  Dosage  MRM  U-MI* | 0.49  0.49  0.49  0.49 | 0.08  0.08  0.09  0.08 | 0  0  0.01  0 | 0  0  0  0 | 0  0  0  0 | 0  0  0  0 |
| 1.2-1.4 | MI SMCFCS  Dosage  MRM  U-MI* | 1  1  1  1 | 0.69  0.69  0.7  0.66 | 0.26  0.27  0.29  0.13 | 0  0  0  0 | 0  0  0  0 | 0.01  0.01  0.01  0 |
| 1.4-1.6 | MI SMCFCS  Dosage  MRM  U-MI* | 1  1  1  1 | 0.97  0.97  0.97  0.95 | 0.85  0.86  0.87  0.57 | 0  0.02  0.02  0 | 0.05  0.05  0.06  0.01 | 0.15  0.14  0.15  0.09 |
| 1.6-1.8 | MI SMCFCS  Dosage  MRM  U-MI* | 1  1  1  1 | 1  1  1  1 | 1  1  1  0.83 | 0.04  0.04  0.04  0 | 0.18  0.17  0.18  0.06 | 0.38  0.37  0.38  0.29 |
| 1.8-2.0 | MI SMCFCS  Dosage  MRM  U-MI* | 1  1  1  1 | 1  1  1  1 | 1  1  1  0.98 | 0.11  0.13  0.13  0.01 | 0.3  0.29  0.3  0.13 | 0.56  0.54  0.55  0.44 |
| 2.0 – 3.0 | MI SMCFCS  Dosage  MRM  U-MI* | 1  1  1  1 | 1  1  1  1 | 1  1  1  1 | 0.62  0.64  0.6  0.25 | 0.82  0.82  0.79  0.63 | 0.91  0.91  0.9  0.86 |
| *U-MI = Unconditional MI | | | | | | | |

**Table S3**: Simulated power with a quantitative trait and n=20,000 observations.

| **Odds ratios** | **Method** | **MAF**$\boldsymbol{\geq}$**0.1**  **Rsq**$\boldsymbol{\geq}$**0.8** | **0.01**$\boldsymbol{\leq}$**MAF<0.1**  **Rsq**$\boldsymbol{\geq}$**0.8** | **0.01**$\boldsymbol{\leq}$**MAF<0.1**  **0.6**$\boldsymbol{\leq}$**Rsq<0.8** | **0.001**$\boldsymbol{\leq}$**MAF<0.01**  **0.3**$\boldsymbol{\leq}$**Rsq<0.6** | **0.001**$\boldsymbol{\leq}$**MAF<0.01**  **0.6**$\boldsymbol{\leq}$**Rsq<0.8** | **0.001**$\boldsymbol{\leq}$**MAF<0.01**  **Rsq**$\boldsymbol{\geq}$**0.8** |
| --- | --- | --- | --- | --- | --- | --- | --- |
| 1-1.2 | MI SMCFCS  Dosage  MRM  U-MI* | 1  1  1  1 | 1  1  1  1 | 1  1  1  0.98 | 0.07  0.11  0.05  0.01 | 0.31  0.33  0.28  0.15 | 0.56  0.56  0.55  0.49 |
| 1.2-1.4 | MI SMCFCS  Dosage  MRM  U-MI* | 1  1  1  1 | 1  1  1  1 | 1  1  1  0.97 | 0.1  0.12  0.06  0.01 | 0.33  0.34  0.29  0.17 | 0.56  0.56  0.55  0.48 |
| 1.4-1.6 | MI SMCFCS  Dosage  MRM  U-MI* | 1  1  1  1 | 1  1  1  1 | 1  1  1  0.97 | 0.09  0.1  0.06  0 | 0.3  0.32  0.27  0.14 | 0.56  0.56  0.55  0.48 |
| 1.6-1.8 | MI SMCFCS  Dosage  MRM  U-MI* | 1  1  1  1 | 1  1  1  1 | 1  1  0.99  0.98 | 0.18  0.19  0.1  0.01 | 0.31  0.32  0.28  0.15 | 0.56  0.56  0.55  0.48 |
| 1.8-2.0 | MI SMCFCS  Dosage  MRM  U-MI* | 1  1  1  1 | 1  1  1  1 | 1  1  1  0.97 | 0.06  0.07  0.04  0 | 0.3  0.31  0.27  0.15 | 0.55  0.55  0.54  0.48 |
| 2.0 – 3.0 | MI SMCFCS  Dosage  MRM  U-MI* | 1  1  1  1 | 1  1  1  1 | 1  1  1  0.98 | 0.1  0.12  0.05  0.02 | 0.33  0.34  0.29  0.16 | 0.57  0.57  0.56  0.49 |
| *U-MI = Unconditional MI | | | | | | | |

**Table S4**: Simulated power with a quantitative trait and n=50,000 observations.

| **Odds ratios** | **Method** | **MAF**$\boldsymbol{\geq}$**0.1**  **Rsq**$\boldsymbol{\geq}$**0.8** | **0.01**$\boldsymbol{\leq}$**MAF<0.1**  **Rsq**$\boldsymbol{\geq}$**0.8** | **0.01**$\boldsymbol{\leq}$**MAF<0.1**  **0.6**$\boldsymbol{\leq}$**Rsq<0.8** | **0.001**$\boldsymbol{\leq}$**MAF<0.01**  **0.3**$\boldsymbol{\leq}$**Rsq<0.6** | **0.001**$\boldsymbol{\leq}$**MAF<0.01**  **0.6**$\boldsymbol{\leq}$**Rsq<0.8** | **0.001**$\boldsymbol{\leq}$**MAF<0.01**  **Rsq**$\boldsymbol{\geq}$**0.8** |
| --- | --- | --- | --- | --- | --- | --- | --- |
| 1-1.2 | MI SMCFCS  Dosage  MRM  U-MI* | 1  1  1  1 | 1  1  1  1 | 1  1  1  1 | 0.45  0.56  0.31  0.15 | 0.79  0.8  0.74  0.52 | 1  1  1  1 |
| 1.2-1.4 | MI SMCFCS  Dosage  MRM  U-MI* | 1  1  1  1 | 1  1  1  1 | 1  1  1  1 | 0.53  0.59  0.35  0.17 | 0.8  0.81  0.75  0.57 | 1  1  1  1 |
| 1.4-1.6 | MI SMCFCS  Dosage  MRM  U-MI* | 1  1  1  1 | 1  1  1  1 | 1  1  1  1 | 0.5  0.54  0.37  0.23 | 0.81  0.82  0.77  0.56 | 1  1  1  1 |
| 1.6-1.8 | MI SMCFCS  Dosage  MRM  U-MI* | 1  1  1  1 | 1  1  1  1 | 1  1  1  1 | 0.43  0.48  0.26  0.11 | 0.78  0.79  0.74  0.53 | 1  1  1  1 |
| 1.8-2.0 | MI SMCFCS  Dosage  MRM  U-MI* | 1  1  1  1 | 1  1  1  1 | 1  1  1  1 | 0.49  0.55  0.33  0.18 | 0.81  0.81  0.75  0.56 | 1  1  1  1 |
| 2.0 – 3.0 | MI SMCFCS  Dosage  MRM  U-MI* | 1  1  1  1 | 1  1  1  1 | 1  1  1  1 | 0.5  0.57  0.32  0.14 | 0.79  0.8  0.74  0.54 | 1  1  1  1 |
| *U-MI = Unconditional MI | | | | | | | |

**Table S5:** Compute times in seconds for analyzing a single SNP with MAF=0.1 across a range of sample sizes and effect sizes for quantitative traits.

| **Effect size** | **Method** | **n=5,000** | **n=10,000** | **n=20,000** |
| --- | --- | --- | --- | --- |
| 0 | MI SMCFCS  Dosage  MRM  U-MI* | 7.259  0.002  0.821  0.264 | 16.600  0.003  1.457  0.462 | 38.817  0.005  4.410  1.164 |
| 0.2 | MI SMCFCS  Dosage  MRM  U-MI* | 7.063  0.002  2.0511  1.280 | 12.792  0.003  3.934  0.449 | 26.434  0.005  9.108  0.969 |
| 0.4 | MI SMCFCS  Dosage  MRM  U-MI* | 6.112  0.002  1.800  2.613 | 14.186  0.003  3.859  0.440 | 26.226  0.005  9.580  1.007 |

*U-MI = Unconditional MI

**Table S6:** Compute times in seconds for analyzing a single SNP with MAF=0.1 across a range of sample sizes and effect sizes for binary traits.

| **Odds Ratio** | **Method** | **n=5,000** | **n=10,000** | **n=20,000** |
| --- | --- | --- | --- | --- |
| 1 | MI SMCFCS  Dosage  MRM  U-MI* | 2.007  0.011  0.578  0.284 | 3.623  0.019  1.259  0.530 | 8.807  0.036  1.978  1.011 |
| 1.2 | MI SMCFCS  Dosage  MRM  U-MI* | 2.768  0.012  5.276  0.874 | 5.318  0.019  5.317  0.829 | 7.477  0.030  7.729  0.936 |
| 1.4 | MI SMCFCS  Dosage  MRM  U-MI* | 2.446  0.012  2.345  0.793 | 4.109  0.020  4.637  0.834 | 12.028  0.033  8.373  0.901 |

*U-MI = Unconditional MI
